# Comprehensive Imaging Evaluation and Staging of Crohn’s Disease: When and Why to Use Intestinal Ultrasound, MRE, or CTE: Current Guidelines and Future Directions

**DOI:** 10.3390/diagnostics16060882

**Published:** 2026-03-16

**Authors:** Francesca Maccioni, Ludovica Busato, Lorenza Bottino, Alessandro Longhi, Alessandra Valenti, Maddalena Zippi, Carlo Catalano

**Affiliations:** 1 Department of Radiological Sciences, Oncology and Pathology, Policlinico Umberto I Hospital, Sapienza University of Rome, 00161 Rome, Italy; 2 Unit of Gastroenterology and Digestive Endoscopy, Sandro Pertini Hospital, 00157 Rome, Italy; maddyzip@yahoo.it

**Keywords:** inflammatory bowel disease (IBD), cross-sectional imaging, intestinal ultrasound (IUS), magnetic resonance imaging (MRI), Crohn’s disease, magnetic resonance enterography (MRE), computed tomography enterography (MRE), anal fistula

## Abstract

Crohn’s disease (CD) is a complex inflammatory bowel disease, defined by chronic transmural inflammation and marked heterogeneity in both anatomical distribution and disease behavior, with potential involvement of any segment of the gastrointestinal tract and multiple phenotypes. Advanced cross-sectional imaging nowadays plays a central role in CD management, reliably assessing both luminal and extraluminal inflammatory manifestations, supporting initial diagnosis, phenotypic characterization, and longitudinal monitoring of disease activity, complications and treatment response. Over the last two decades, Intestinal Ultrasound (IUS), MR Enterography (MRE), and Computed Tomography Enterography (CTE) have become central components of the diagnostic pathway. MRE has emerged as the most comprehensive, radiation-free modality for evaluating intestinal extent, inflammatory activity, and complications in Crohn’s disease. Multiparametric MRE, combining T2-weighted imaging, contrast-enhanced sequences, diffusion-weighted imaging, and cine acquisitions, enables a real “Crohn’s disease staging”, namely a thorough evaluation of the transmural inflammation, of fibrotic and fistulizing lesions in the small and large bowel, as well as in the perianal region. IUS provides a dynamic, widely accessible, safe and repeatable imaging technique that is particularly well suited for tight-monitoring strategies, early assessment of therapeutic response, and routine follow-up, especially in experienced centers. Notably CTE, despite concerns related to cumulative ionizing radiation exposure, remains indispensable in acute clinical settings owing to its rapid acquisition, broad availability, and high diagnostic accuracy for detecting abscesses, perforation, and bowel obstruction. Combined, these three modalities offer a complementary and patient-tailored framework for optimal CD management. This review outlines the pathological complexity of Crohn’s disease, traces the evolution of imaging approaches, and provides a comparative overview highlighting the specific strengths and limitations of each modality.

## 1. Introduction

Inflammatory Bowel Disease (IBD) is a group of chronic inflammatory conditions of the gastrointestinal (GI) tract, mainly Crohn’s disease (CD) and Ulcerative Colitis (UC). It occurs when the immune system mistakenly attacks healthy intestinal tissue, leading to persistent inflammation and structural damage to the bowel. Unlike UC, which is confined to the colon and in which inflammation affects only the inner mucosal layer of the intestinal wall, CD can involve any segment of the gastrointestinal tract, from the mouth to the anus; furthermore, the inflammation is transmural, affecting all layers of the intestinal wall.

Cross-sectional imaging plays a pivotal role in evaluating IBD and particularly CD, owing to its complex nature, heterogeneous involvement of the small and large bowel, variable transmural inflammation and fibrosis, and increased risk of severe complications. As endoscopic techniques primarily assess mucosal involvement, cross-sectional imaging is essential to capture the inflammatory involvement of deeper layers of the bowel wall and the surrounding mesenteric environment. Overall, the evaluation of CD has evolved considerably over the past two decades: the progressive shift from traditional fluoroscopic examinations to high-resolution cross-sectional imaging techniques reflects the evolving landscape of IBD diagnosis, monitoring, and management [1]. This evolution highlights both the growing understanding of the disease’s complexity and the continuous technological development of imaging modalities [2]. Today, radiology is no longer limited to a supportive role alongside endoscopy but plays a central role in the diagnosis, disease staging, treatment planning, and long-term monitoring. Among the available radiological techniques, cross-sectional imaging modalities, particularly Magnetic Resonance Enterography (MRE), Intestinal Ultrasound (IUS), and Computed Tomography Enterography (CTE), have become integral to the comprehensive evaluation and management of Crohn’s disease, each offering distinct advantages and limitations and being optimally suited to specific diagnostic and clinical scenarios. This panoramic review aims to provide a clinically oriented, guideline-based overview of the complementary roles of IUS, MRE and CTE in the management of Crohn’s disease. It highlights the respective strengths and limitations of each technique, as well as their optimal indications in different clinical scenarios. Particular attention is given to comprehensive disease staging and the evolution of international imaging recommendations.

## 2. Crohn’s Disease: Multiple Phenotypes and Pathological Complexity

Crohn’s disease is arguably the most anatomically and pathologically heterogeneous form of IBD. It may involve any portion of the gastrointestinal tract, from the esophagus and stomach through the entire small bowel, and encompassing all colonic segments, including rectum and anal canal. Although over 80% of patients experience terminal ileal involvement, the site and extent of the disease remain unpredictable: lesions may be single or multiple, vary greatly in length and distribution, and may change over time [1,2,3].

A hallmark of Crohn’s disease is the transmural inflammation, which extends from the mucosa to the serosa, frequently accompanied by a perivisceral inflammatory reaction. Mesenteric fat hypertrophy, locoregional oedema, lymphadenopathy, and mesenteric vascular alterations are commonly observed, particularly in active phases. In more advanced or complicated disease, fistulas (entero-enteric, entero-vesical, entero-cutaneous), abscesses, and phlegmons may develop, which may extend to adjacent anatomical structures such as the psoas or lumbar musculature [4].

From a morphological perspective, Crohn’s disease may present with predominantly inflammatory, fibrostenotic or penetrating phenotypes, however mixed phenotypes are, frequently observed, particularly in longstanding disease [3]. Because a complete and durable regression of lesions is rare, patients often cycle through periods of remission and relapse, progressively accumulating structural alterations such as strictures, adhesions, and deformation of intestinal loops. This cumulative bowel damage underscores the necessity for advanced imaging modalities, capable of evaluating both luminal and extraluminal components of the disease, at any level of the GI tract [4].

### The Need for a Comprehensive CD Assessment

Given the multifaceted pathological manifestations of Crohn’s disease, a thorough evaluation requires an integrated imaging approach. Comprehensive Crohn’s disease assessment or “staging” represents a complex diagnostic process encompassing several key components:

- First, initial diagnosis, which may be challenging in differentiating Crohn’s disease from infectious enteritis or ulcerative colitis, particularly in the presence of CD colonic involvement;
- Second, assessment of disease extent, including the number, length, and anatomical distribution of lesions in the small and large bowel, which may vary considerably among patients;
- Third, quantification of inflammatory activity at the level of each affected bowel segment;
- Fourth, characterization and quantification of fibrotic changes within the bowel wall;
- Fifth, identification of transmural and extra-intestinal complications, such as fistulas, abscesses and phlegmons;
- Furthermore, evaluation and staging of perianal disease;
- Finally, in long-standing disease, assessment of cumulative bowel damage and surveillance for dysplasia or precancerous lesions.

## 3. Evolution of Imaging in Crohn’s Disease

No single imaging modality can comprehensively address all these aspects in every patient. Instead, the optimal diagnostic strategy relies on the complementary use of different imaging techniques, tailored to specific clinical questions, patient characteristics, and practical considerations.

In order to better understand the current role of imaging modalities in the management of Crohn’s disease, it is helpful to briefly outline their historical development and the progressive shift towards radiation-free and multiparametric approaches.

### 3.1. From Barium Studies to Cross-Sectional Techniques

In the late 1990s and early 2000s, Rioux described the “puzzling” imaging world of Crohn’s disease, highlighting the need to combine conventional barium examinations with cross-sectional methods such as ultrasound and CT to achieve a complete evaluation [5]. Oral contrast fluoroscopy was particularly useful for detecting small-bowel disease, identifying strictures and fistulas. Barium follow-through examinations provided good visualization of mucosal irregularities, strictures, and fistulas in the small bowel, but lacked extraluminal assessment. Meanwhile, ultrasound became an important tool for targeted assessment of the terminal ileum, especially when combined with color Doppler to evaluate mural vascularity and inflammation. CT, on the other hand, quickly emerged as the reference technique for complicated disease, particularly for detecting perforations, abscesses, and complex fistulas. Nuclear medicine studies complemented this approach by helping confirm abscesses or regions of significant inflammation. A major turning point occurred in the early 2000s with the increasing availability and refinement of MRE. The 2013 ECCO–ESGAR guidelines marked the first official recognition of MRE as a central imaging tool recommended for the assessment of most Crohn’s disease manifestations. In the subsequent ECCO–ESGAR guidelines published in 2019 and 2025 [2,3], MRI was variably integrated with ultrasound and CT in order to assess different aspects of Crohn’s disease, as discussed in detail in Section 5.

### 3.2. MR Enterography: A Multiparametric, Radiation-Free Tool for Comprehensive Crohn’s Disease Staging

The introduction and progressive refinement of MR Enterography in the early 2000s represented a major turning point in Crohn’s disease imaging [6,7,8]. By 2013, the ECCO–ESGAR consensus formally recognized MRE as a key modality for evaluating disease extent, inflammatory activity, complications, and treatment response. Its radiation-free nature and multiparametric capabilities progressively established MRE as the reference technique for comprehensive transmural assessment [8,9,10]. Furthermore, High-Resolution (HR) MRI also represents the gold standard for diagnosing and staging perianal fistulas. The final paragraph of this review is therefore dedicated to a focused discussion on HRMRI, highlighting its role in accurate anatomical delineation of anal and perianal fistulas, disease classification and staging, and clinical decision-making.

Unlike CT, MRE avoids ionizing radiation and is therefore particularly suitable for younger patients who require lifelong follow-up. Unlike IUS, MRE simultaneously provides a panoramic and accurate view of the entire small bowel, of the entire colon and anorectal region. One of the principal strengths of MRE lies in its intrinsic multiparametric nature. By integrating complementary morphological and functional sequences, MRE enables comprehensive characterization of both Crohn’s disease activity and behaviour. Its central role is largely attributable to its ability to provide a panoramic, high-contrast evaluation of the entire abdominopelvic cavity without exposing patients to ionizing radiation. These advantages are particularly relevant given the chronic relapsing nature of Crohn’s disease and the young age at diagnosis for many patients, which makes repeated imaging a lifelong requirement [11,12].

Multiparametric MRE therefore combines a series of complementary sequences, each providing distinct and synergistic diagnostic information. In clinical practice, approximately 1.5 L of polyethylene glycol–based oral contrast is administered to achieve adequate small-bowel distension. MR enterography is performed after about 30 min associating several different sequences to achieve both morphological and functional information, usually including i.v. administration of an antiperistaltic agent and the intravenous injection of a gadolinium-based contrast medium (cyclic Gd-molecule configuration), using slightly different protocols if working with a 1.5 or a 3 Tesla Magnet, as summarized in Table 1.

**Figure 1:**
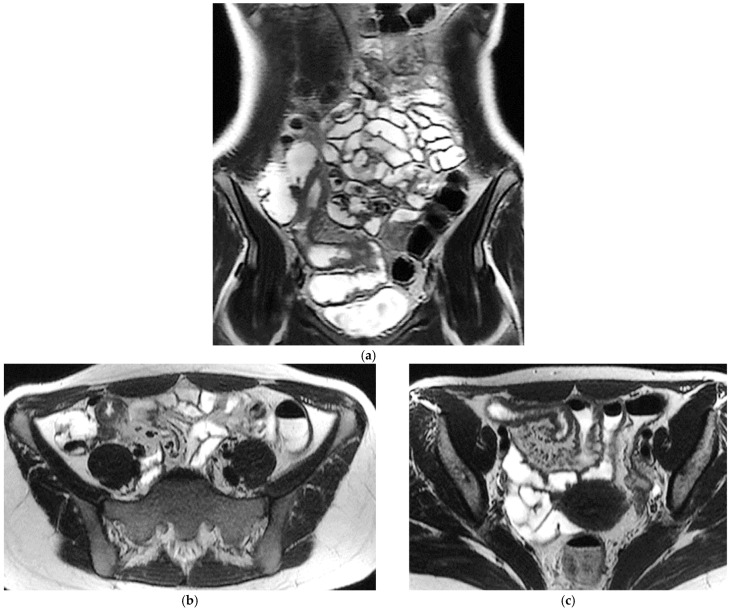

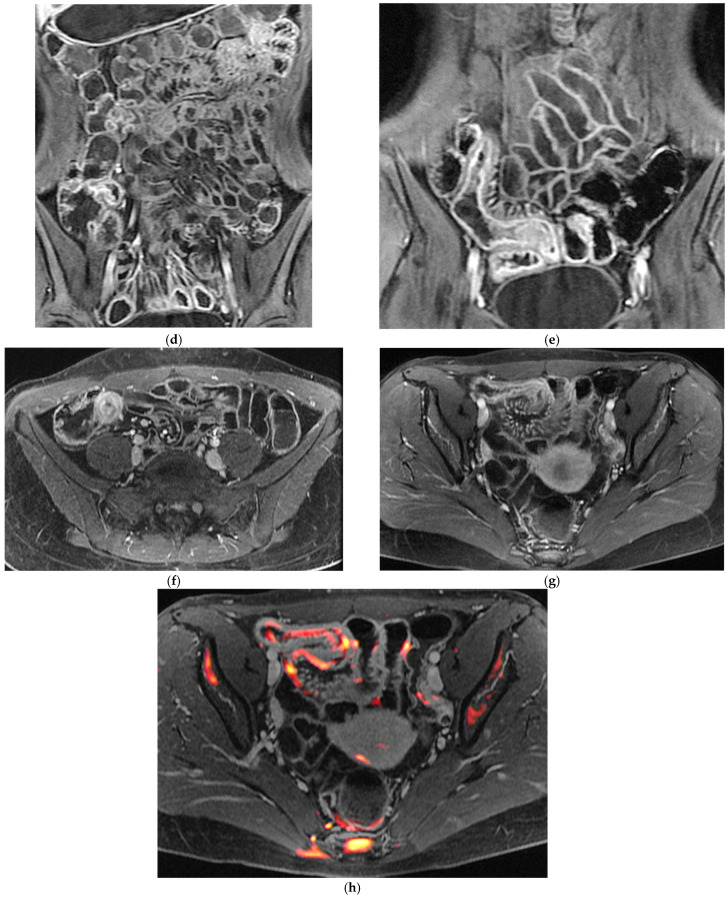
20-year-old female patient with typical Crohn’s disease of the terminal ileum, evaluated with a 3T Magnet, following oral ingestion of 1500 cc of PEG solution. (**a**): Coronal T2w images obtained with breath-hold acquisitions show diffuse inflammatory thickening of the last ileal loop exceeding 15 cm in length. Note how the remaining loops of the small bowel wall appear normal in contrast with the affected ileal loop. (**b**,**c**): axial T2 w images showing pathological thickening of the last ileal loop. (**d**,**e**): coronal T1-weighted scan after gadolinium injection, showing marked enhancement of the ileocecal valve and terminal ileum wall. In particular, an increased mucosa enhancement is clearly visible in the terminal ileum, well distinguishable from the normal mucosa of the remaining ileal and jejunal loops. The terminal ileum is affected for at least 15–20 cm; local mesenteric hyperaemia is also observed. The entire small bowel is fully displayed (**d**), including jejunal and ileal loops, which appear normal with respect to the affected terminal ileum. (**f**,**g**): Axial Gd-enhanced T1-weighted images show marked concentric wall thickening and enhancement of the terminal ileum with associated mesenteric hyperaemia, due to severe inflammatory involvement. (**h**): Post-processing “fusion” image, in which the T2 image is merged with the post-contrast T1 image, highlighting the pathological ileal loop in 20-year-old female patient with Crohn’s disease.

T2-weighted sequences (Figure 1a–c) are fundamental for a comprehensive morphological assessment of Crohn’s disease on both axial and coronal planes. They allow accurate definition of disease location, longitudinal extent, and severity, including evaluation of bowel wall thickening, luminal narrowing, and the presence of penetrating complications such as fistulas and abscesses at any level of the small and large bowel. These sequences are also essential for assessing extraintestinal manifestations of disease, including inflammatory changes of the surrounding mesenteric fat. Beyond structural assessment, T2-weighted imaging plays a pivotal role in the detection of inflammatory activity through the identification of mural and mesenteric oedema. In particular, Fat-Suppressed T2-weighted sequences enhance the conspicuity of oedematous tissue with a higher T2-signal, and currently represent the only imaging technique capable of directly demonstrating oedema at the level of the intestinal wall, mesenteric lymph nodes, and mesentery. Oedema is the hallmark of active inflammation, reflecting increased vascular permeability and interstitial fluid accumulation [6,12,13,14,15,16]. As such, it provides information that is complementary to mural hypervascularization observed on contrast-enhanced sequences, contributing to a more integrated and comprehensive assessment of disease activity [15,16]. Pre- and post-gadolinium–enhanced T1-weighted sequences enable detailed assessment of mucosal and transmural enhancement patterns (Figure 1d–g), which reflect bowel wall vascularity and the degree of inflammatory activity. These sequences are essential for evaluating not only the affected bowel segments, but also associated inflammatory changes in the mesentery and regional lymph nodes.

In addition, dynamic assessment of Gd-enhancement over time provides clinically relevant information in patients with Crohn’s disease. In the setting of active inflammatory disease, affected bowel segments typically demonstrate early and intense mural enhancement during the arterial phase, approximately 20 to 30 s after intravenous contrast administration, reflecting hyperemia and increased vascular permeability. Conversely, in predominantly fibrotic (fibrostenotic) disease, Gd-enhancement tends to be more gradual and progressive, with peak mural enhancement observed in the delayed phases, approximately 3–7 min after contrast injection. This delayed pattern is consistent with reduced vascularity and a greater proportion of fibrotic tissue within the bowel wall [17,18,19].

Therefore, temporal evaluation of contrast enhancement plays a key role in differentiating predominantly inflammatory from mixed or fibrostenotic phenotypes, thereby supporting more tailored and phenotype-driven therapeutic decision-making.

Diffusion-weighted imaging (DWI) provides important functional information by probing the microscopic mobility of water molecules within tissues. In Crohn’s disease, restricted diffusion is a well-established imaging biomarker of active inflammation, reflecting increased cellularity, inflammatory cell infiltration, and reduced extracellular space. Several studies have demonstrated a strong correlation between DWI signal intensity, apparent diffusion coefficient (ADC) values, and endoscopic or histopathological markers of disease activity, supporting its role as a non–contrast-based surrogate of inflammation and its potential utility in treatment monitoring, especially in patients with contraindications to gadolinium-based contrast agents [18,19,20,21]. Furthermore, Diffusion-weighted imaging (DWI) abnormalities can also reflect fibrotic changes, thereby extending the utility of this sequence beyond the detection of pure inflammation [12].

Cine-MRI sequences further enhance functional assessment by capturing real-time bowel motility. These sequences, typically acquired using dynamic T2-weighted imaging or balanced steady-state free-precession techniques such as TRUFI (True Fast Imaging with Steady-State Precession), allow continuous visualization of intestinal peristalsis. Cine imaging is invaluable for differentiating fixed fibrostenotic strictures, which demonstrate persistently reduced or absent motility, from transient luminal narrowing caused by spasm or peristaltic contraction [22,23]. In addition, cine-MRI can reveal subtle motility abnormalities in upstream or downstream bowel segments that may not be apparent on static images, contributing to a more comprehensive evaluation of disease burden and functional impairment. When integrated with morphological and contrast-enhanced sequences, cine-MRI strengthens the multiparametric approach of MRE, improving phenotypic characterization and supporting tailored therapeutic strategies.

The capability of MRE to acquire images in multiple planes with high spatial and contrast resolution further allows refined assessment of both mural and extramural disease components, including mesenteric involvement and penetrating complications. Because images are routinely obtained both in axial and coronal planes, MRE permits an accurate localization of bowel lesions, detailed morphological characterization, and comprehensive mapping of disease extent across the entire gastrointestinal tract.

Technologically, MRE is also a rapidly evolving modality. Continuous advances in sequence design, acquisition speed, motion correction strategies, and functional imaging parameters have progressively improved image quality and diagnostic performance [20]. This ongoing technical evolution ensures that MRE remains at the forefront of non-invasive imaging for the assessment of transmural inflammation in Crohn’s disease. As a result of these advantages, MRE has progressively established itself as the reference imaging modality for a comprehensive evaluation of Crohn’s disease, particularly when full staging or re-staging is required. Its central role is attributable not only to its multiparametric approach but also to its ability to provide a panoramic, high-contrast evaluation of the entire abdominopelvic cavity without exposing patients to ionizing radiation [20]. These features are especially relevant given the chronic relapsing nature of Crohn’s disease and the young age at diagnosis for many patients, which necessitate repeated imaging over a lifetime.

**Figure 2:**
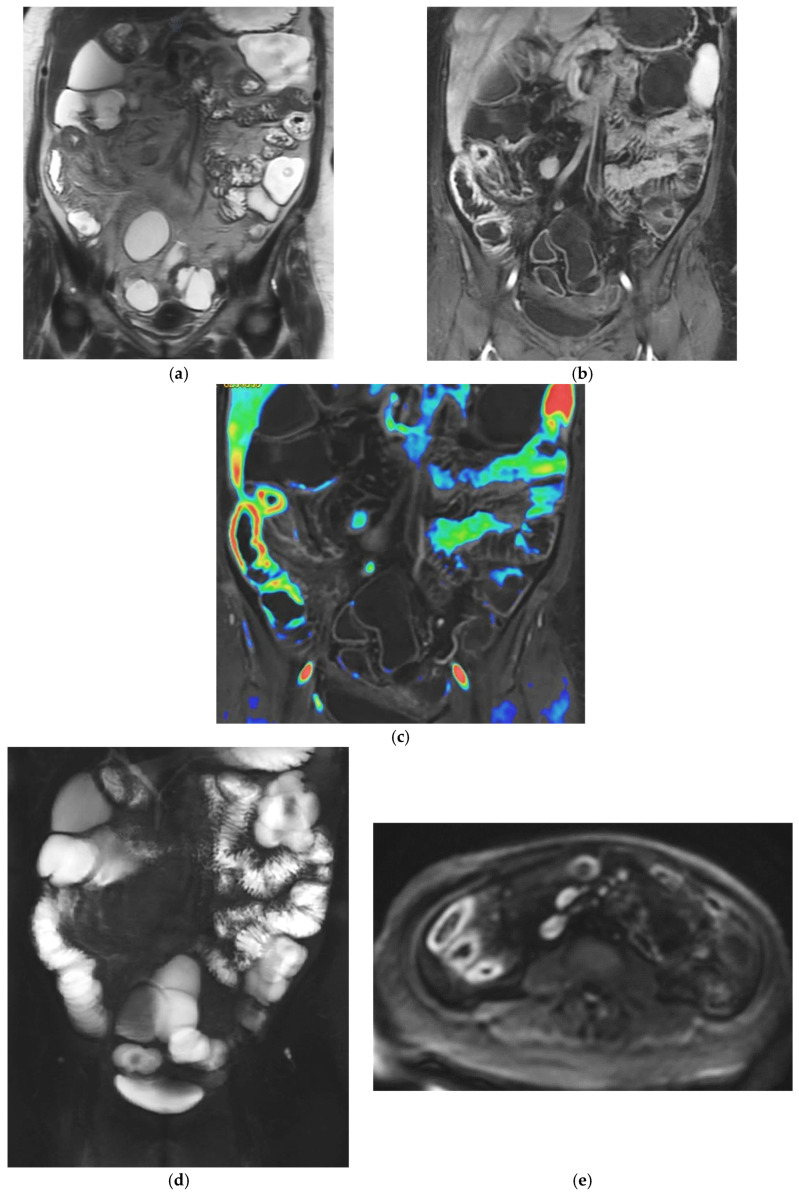
47-year-old female patient with recurrent ileal Crohn’s disease following resection. (**a**) Coronal T2-weighted image demonstrating recurrent ileal Crohn’s disease at the anastomotic site, characterized by marked bowel wall oedema. (**b**) Corresponding axial T1-weighted image after gadolinium administration showing intense mural hyperenhancement of the affected ileal loop. (**c**) Post-processed color-coded map derived from the T1-weighted gadolinium-enhanced sequence, with areas of maximal enhancement highlighted in red. (**d**) Single-shot fast spin-echo sequence with follow-through–like appearance, clearly depicting the lumen of both normal and pathological small-bowel loops. (**e**) Axial diffusion-weighted imaging (DWI) showing marked diffusion restriction within the diseased ileal segment.

This combination of structural and functional information makes MRE particularly powerful in assessing CD inflammatory activity. Increased mural thickness, hyperintensity on T2-weighted imaging, layered or transmural enhancement after contrast administration and restricted diffusion are features that correlate closely with endoscopic markers of disease severity [6,7,8,9,10,11,12,13,14,15,16,17,18,19,20]. Indeed, these imaging markers form the basis of validated scores such as the Magnetic Resonance Index of Activity (MaRIA), which is now widely accepted as a reliable surrogate for mucosal inflammation in both the ileum and colon [11,12,13,17,19,20,21]. CD activity MRI features can be further enhanced using Fusion Imaging and post-processing techniques, which can synthesize and highlight Gd-enhancement, T2-weighted and DWI at the level of the affected bowel loops (Figure 1h and Figure 2c).

A major advantage of MRE, which strongly influences clinical decision-making, is its capacity to characterise strictures with exceptional detail. The ability to distinguish inflammatory from fibrotic narrowing relies on the multiparametric information inherent to the technique: active inflammatory strictures typically demonstrate high T2 signal, prominent or layered enhancement, and restricted diffusion, whereas fibrotic strictures often appear as low-signal, homogeneous, and relatively non-enhancing segments with preserved diffusion characteristics [12,20,21]. This distinction is critical, as treatment of inflammatory strictures favours medical escalation—including biologics—whereas fibrotic strictures often require endoscopic or surgical intervention.

MRE is equally valuable in the detection of penetrating complications, an area where its sensitivity rivals or surpasses that of CT [14]. MRE is particularly effective in identifying entero-enteric and entero-colonic fistulas, subtle sinus tracts, intramural or mesenteric abscesses, and inflammatory phlegmons. These complications carry significant prognostic weight, often predicting a more aggressive disease course and influencing therapeutic strategies. Because MRE enables both mural and extramural structures comprehensively, it provides a more complete assessment of disease behaviour than endoscopy alone [9] (Figure 1 and Figure 2). The diagnostic performance of MRE has been validated in several prospective comparative studies [5,6,7,8,9,10,11,12,13,14,15,16,17], such as the multicenter METRIC trial, showing a sensitivity of approximately 97% for detecting the presence of small-bowel Crohn’s disease and around 80% for defining disease extent [23]. In the same study, IUS demonstrated a slightly lower sensitivity for the detection of small-bowel disease (approximately 92–94%), a specificity comparable to that of MRE (both exceeding 90%), although the accuracy in defining the full extent of disease inferior to MRE (70%). Overall, while both modalities showed high diagnostic performance, MRE provided a superior sensitivity for disease presence and extent, particularly in the proximal small-bowel segment [23]. MRE also showed high accuracy in identifying active inflammation and extramural complications, consolidating its role as the reference cross-sectional modality for comprehensive small-bowel evaluation [8].

Several MRI-based activity indices have been developed to standardize the assessment of inflammatory burden in Crohn’s disease and to provide reproducible metrics for clinical practice and research [11,13,19,20,21,22,23,24,25,26,27,28]. The Magnetic Resonance Index of Activity (MaRIA) is the most widely validated score and incorporates mural thickness, relative contrast enhancement, presence of oedema on T2-weighted imaging, and ulceration to quantify disease activity in individual bowel segments [11,13,19,20,21,22,23,24,25,26,27,28]. The MaRIA score correlates strongly with endoscopic severity and is widely used as an imaging surrogate for mucosal inflammation, particularly within treat-to-target strategies [11]. It has demonstrated a robust correlation with established endoscopic severity indices, with reported area under the curve (AUC) values frequently exceeding 0.80–0.85 for the detection of active inflammation. These findings support its role as an objective imaging biomarker in treat-to-target approaches. The simplified MaRIA score was then developed to improve feasibility in routine practice while maintaining strong correlation with endoscopic activity [25]. Both the original and simplified MaRIA scores demonstrate a strong association with endoscopic indices, particularly the SES-CD, which remains a validated reference standard for mucosal healing. However, MRI-based indices provide complementary information by assessing transmural inflammation and complications, beyond mucosal involvement and beyond endoscopic evaluation. In this context, MaRIA may serve as a non-invasive surrogate of endoscopic activity while also capturing transmural healing, a therapeutic endpoint that extends beyond mucosal healing alone. Transmural healing is an emerging and more ambitious therapeutic endpoint that extends beyond mucosal healing alone and reflects the evolving concept of deep remission in Crohn’s disease management; multiparametric magnetic resonance enterography (MRE) represents one of the most comprehensive non-invasive tools currently available to assess this target [29].

The Crohn’s Disease Magnetic Resonance Index (CDMI) represents an alternative scoring system that emphasises mural thickness, T2 hyperintensity, and perimural inflammatory changes, offering a slightly simplified framework that may be more feasible in routine practice while maintaining good correlation with histologic and clinical markers of activity [13,25,26]. The Clermont score, derived from diffusion-weighted imaging (DWI), integrates the apparent diffusion coefficient (ADC) with mural thickness and relative contrast enhancement, thereby complementing conventional parameters with a functional biomarker of inflammatory cellularity [13,18,25,26,27,28]. Because DWI is sensitive to restricted diffusion associated with active inflammation, the Clermont score has shown high accuracy in differentiating active from quiescent disease and may be particularly valuable when gadolinium administration is contraindicated [18,19]. Together, these scoring systems enhance the objectivity of MRI interpretation, facilitate longitudinal monitoring, and support the use of MRI as a reproducible endpoint in clinical trials and clinical decision-making (Table 2).

Last, MRE represents the imaging modality with the greatest potential for integration with artificial intelligence (AI) in Crohn’s disease. The multiparametric nature of MRE, combining morphological sequences with functional and quantitative information such as diffusion-weighted imaging and contrast enhancement, provides a rich dataset for machine learning applications. Preliminary studies have shown that AI-based approaches can achieve performance comparable to expert readers in automated bowel segmentation, detection of inflamed segments, and quantification of disease activity through radiomics and deep learning models [30,31,32]. In addition, advanced radiomic pipelines allow extraction of high-dimensional quantitative features related to texture, signal heterogeneity, and enhancement kinetics, which may capture subtle inflammatory and fibrotic changes not appreciable by visual assessment alone. Deep learning architectures, particularly convolutional neural networks and transformer-based models, have also been explored for automated differentiation between inflammatory and fibrostenotic strictures, a clinically relevant distinction that directly influences therapeutic decision-making [31,32].

Emerging evidence further suggests that AI models trained on longitudinal MRE datasets may help predict treatment response, identify patients at risk of disease progression, and estimate the likelihood of penetrating or stricturing complications. Integration of imaging features with clinical, endoscopic, and laboratory parameters within multimodal AI frameworks may enhance risk stratification and support personalized management strategies. However, despite these promising developments, current evidence remains largely based on retrospective, single-center studies with limited external validation. Standardization of acquisition protocols, harmonization of radiomic features, transparent reporting, and prospective multicenter validation are essential steps before routine clinical implementation. In the near future, AI-enhanced MRE may enable objective, reproducible assessment of disease activity, prediction of treatment response, and longitudinal monitoring, thereby reducing interobserver variability and improving workflow efficiency. These advances establish MRE as a key platform for precision imaging in Crohn’s disease [32].

Beyond its diagnostic accuracy and potential, MRE offers several practical advantages. The absence of ionising radiation makes it suitable for repeated follow-up, which is essential for chronic diseases such as Crohn’s. Its panoramic evaluation of the small bowel is unmatched, and its capacity to assess soft tissues, mesentery, and extra-intestinal complications in a single examination is unique among available modalities.

However, MRE is not without limitations. It requires longer acquisition times compared with CT, and patient cooperation, including multiple breath-holding acquisitions and tolerance of luminal distension, both essential for optimal image quality. Motion artefacts from peristalsis may necessitate the use of antispasmodic agents. Furthermore, despite growing availability, MRE remains more resource-intensive than ultrasound, both in terms of cost and scanner time, and may not be immediately accessible in all clinical settings.

In summary, MRE is a safe and complex imaging procedure which provides an incomparable combination of anatomical detail and functional information, making it the most comprehensive non-invasive tool for the assessment of Crohn’s disease across its inflammatory, stricturing, and penetrating manifestations.

### 3.3. Intestinal Ultrasound (IUS): A Clinical-Radiological Crohn’s Disease Monitoring Tool

Renewed interest in intestinal ultrasound (IUS) reflects a broader shift in Crohn’s disease management toward tight-control and treat-to-target strategies, in which rapid, repeated assessment of disease activity is essential. Once considered mainly a complementary or supportive technique, IUS has progressively become a key clinical–radiological tool, particularly in large gastroenterology centers with integrated imaging expertise. Its growing importance is attributable not only to technical refinement, but also to its intrinsic clinical strengths: it is non-invasive, radiation-free, cost-effective, easily repeatable, and capable of providing immediate, real-time information at the bedside [33,34,35,36,37,38]. Meta-analyses have reported that intestinal ultrasound achieves sensitivity ranging from approximately 84% to 92% and specificity between 88% and 96% for detecting small-bowel Crohn’s disease, particularly in expert centers. The addition of Doppler and contrast-enhanced ultrasound further improves assessment of inflammatory activity and abscess characterization. Another relevant factor is the increasing dissemination of this technique not only among radiologists but also among gastroenterologists. These characteristics align exceptionally well with the needs of chronic inflammatory diseases, in which dynamic monitoring and frequent reassessment are essential.

**Figure 3:**
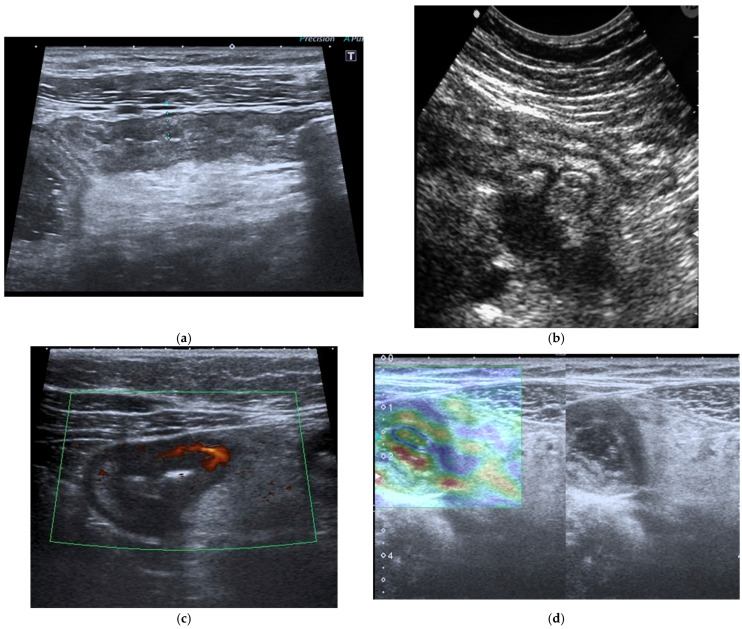
(**a**) IUS of the ileocecal region shows wall thickening (6 mm) of the terminal ileal loop with associated cecal wall thickening in a patient affected by CD. (**b**) Complicated perforating CD: IUS shows marked wall thickening of an ileal loop with multiple associated mesenteric abscesses. (**c**) Patients with active CD: ultrasound axial scan of the affected ileal loop, shows wall thickening and increased vascularity at Color Power Doppler. (**d**) Elastosonography of an ileal loop affected by CD shows marked wall thickening and mild fibrosis (yellow); please note that blue-green color is lack of fibrosis, whereas yellow and red is mild to severe fibrosis.

The fundamental strengths of IUS derive from its ability to visualize bowel wall thickness, mural stratification, and peristaltic activity with high temporal resolution. Bowel wall thickening represents one of the most robust markers of inflammatory activity and, when combined with color or power Doppler evaluation, ultrasound becomes a sensitive tool for assessing mural hypervascularity, considered a surrogate marker of active inflammation [33,34,35,36,37,38] (Figure 3a–c). The Milan Criteria were recently developed by the International Bowel Ultrasound Group (IBUS) and included in the latest guidelines to standardize IUS assessment of inflammatory bowel disease [38]. Contrast-enhanced ultrasound (CEUS) allows dynamic evaluation of microvascular perfusion, enabling a more refined analysis of transmural enhancement patterns. CEUS is useful for early assessment of therapeutic response as well as for the identification and characterization of abscesses. Another promising development is ultrasound elastography, which may be useful in distinguishing inflammatory from fibrotic strictures, a diagnostic challenge traditionally dominated by MRE [38] (Figure 3d).

However, the main limitations of IUS should be acknowledged. The major limitation of this technique lies in its strong operator dependence. Diagnostic performance is highly influenced by variations in training, experience, and equipment. Adequate expertise usually requires exposure to a high volume of cases with systematic correlation to more robust imaging modalities, a condition typically met only in centers of excellence. Another limitation is the poor reproducibility of the examination, its extreme focality, and the lack of panoramic assessment, which makes a complete and reliable evaluation of the small bowel impossible. Furthermore, image acquisition depends exclusively on the operator’s manual skill, which is therefore not reproducible or standardized, as in CT or MRI. Moreover, the diagnostic accuracy is strongly dependent on patient body habitus and on the presence of intestinal gas, with the inability to evaluate gas-distended bowel loops and retroperitoneal regions. Body habitus may reduce acoustic penetration, limiting evaluation in obese patients; certain bowel segments, particularly deep pelvic loops or proximal small-bowel segments, may not be adequately visualized [33,36].

One of the most interesting aspects of IUS in gastroenterological practice is its suitability for frequent monitoring, making it a natural imaging complement to treat-to-target strategies, particularly in younger and pediatric patients. Unlike MRE or CT, which require logistical coordination, IUS can be repeated as often as necessary, allowing proactive therapeutic adjustments based on early identification of subclinical disease activity. This is particularly useful in the management of patients receiving biologic therapies, in whom early changes in bowel wall thickness or vascularity often correlate with long-term treatment outcomes. A potential source of bias may arise from “internal” rather than independent or external assessment of treatment response, since ultrasound is often performed by the same gastroenterologist who prescribes the biologic therapy, or by a gastroenterologist within the same clinical team. Thus, what constitutes a strength may also represent a potential bias. Further evidence is needed to confirm this potential limitation and to better clarify its impact on outcome assessment.

For these reasons, although IUS plays a valuable role in routine monitoring and rapid assessment, it is generally insufficient for complete disease mapping at diagnosis or in complex cases requiring a comprehensive overview. In such scenarios, MRE remains the reference imaging modality [12,14,20,21,26].

### 3.4. Computed Tomography/CT Enterography (CTE): Rapid, Powerful Modality for Emergency Crohn’s Disease Evaluation

Although the long-term management of Crohn’s disease increasingly favors radiation-free imaging approaches, computed tomography remains an essential tool, particularly in the acute setting where rapid diagnosis is critical. CT’s speed, broad availability, and excellent spatial resolution make it the cornerstone imaging modality in emergency departments worldwide, where patients often present with severe abdominal pain, fever, or signs suggestive of abscess formation, perforation, or high-grade obstruction.

The diagnostic strengths of CT in Crohn’s disease are primarily related to its superior detection of extramural and transmural complications.

CT is unmatched in demonstrating extraluminal air, fluid collections, and the pattern and extent of inflammatory changes in the surrounding mesentery, when perforation is suspected, whether free perforation or a contained microperforation. Mesenteric abscesses, phlegmons, and complex fistulous tracts are readily identified, and CT’s rapid acquisition allows diagnosis even in clinically unstable patients who may not tolerate longer imaging protocols. In cases of suspected obstruction, CT excels in determining the site and severity of luminal narrowing, detecting pre-stenotic dilation, and identifying transition points-information that is crucial for guiding urgent surgical or endoscopic intervention [39,40,41].

**Figure 4:**
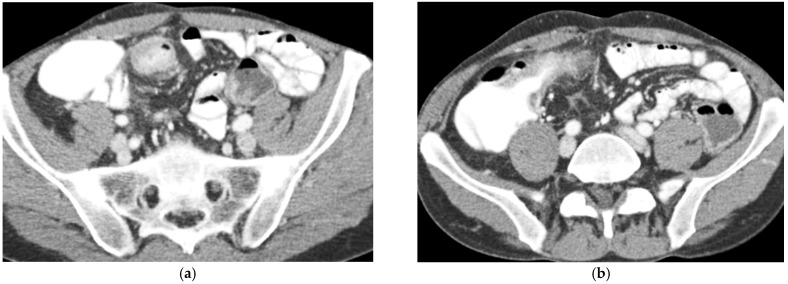

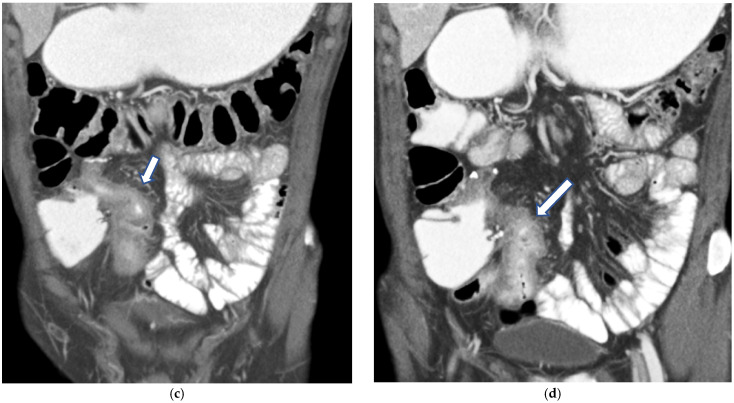
(**a**,**b**) Axial CT scan in the venous phase after administration of positive oral contrast medium (Gastrografin) showing thickened terminal ileum in a 65-year-old patient with Crohn’s disease. (**c**,**d**) Coronal CT scan in the venous phase after administration of positive oral contrast medium (Gastrografin) showing thickened terminal ileum (arrows) in a 65-year-old patient with Crohn’s disease.

**Figure 5:**
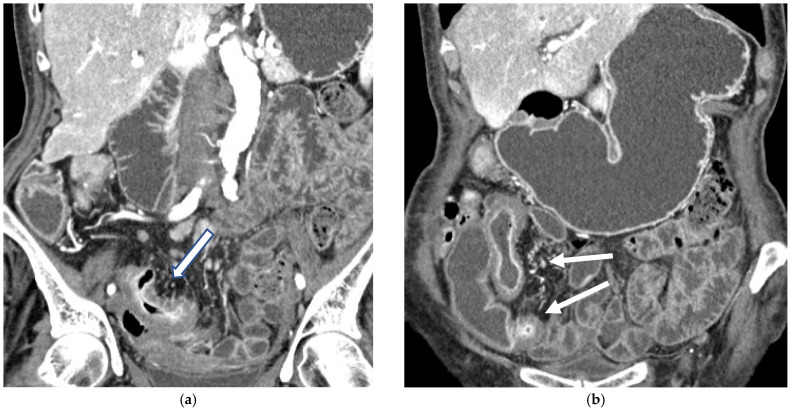

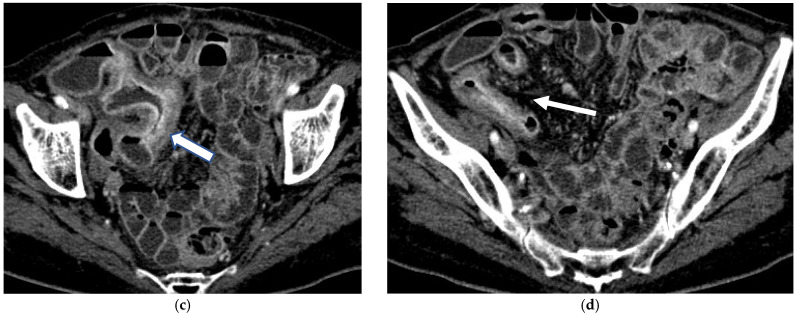
(**a**,**b**) Coronal CT scan in the venous phase after administration of negative oral contrast medium (PEG-solution) showing thickened ileal loops (arrows) in an 85-year-old female patient with Crohn’s disease. (**c**,**d**) axial CT scan in the venous phase after administration of negative oral contrast medium (PEG-solution) showing thickened ileal loop (arrows) in an 85-year-old female patient with Crohn’s disease.

CT Enterography (CTE) is a dedicated technique that can be performed using either neutral (water macrogol solutions) or positive (iodinated) oral contrast for optimal luminal distention, thereby enhancing the modality’s utility in elective outpatient evaluation, shown on Figure 4 and Figure 5. CTE performed with positive oral contrast improves visualization of the entire small bowel lumen and interloop relationships, and is particularly useful in complex disease associated with adhesions and fistulas (Figure 4a–d and Figure 5a–d). On the other hand, CTE performed with negative oral contrast enables assessment of mucosal hyper-enhancement, wall thickening, and inflammatory changes (Figure 5). However, although CTE can approximate much of the mural and extramural information obtained with MRE, it lacks the functional imaging capabilities of MRI and cannot distinguish with confidence between inflammatory and fibrotic strictures. For this reason, CT is generally reserved for situations in which MRE is unavailable or contraindicated, or when specific luminal abnormalities, such as unexplained focal thickening or suspected small-bowel polyps, require rapid clarification [20,21,39,40,41].

The limitations of CT/CTE in Crohn’s disease are inseparable from its greatest challenge: ionizing radiation. Young patients with IBD are particularly vulnerable to the cumulative effects of repeated CT scans, and concerns regarding radiation exposure have prompted significant efforts to limit CT use outside emergency settings. Even with dose-reduction strategies, CT is not favored for routine follow-up or treat-to-target intervals. Furthermore, CT lacks the multiparametric functional detail provided by MRI and the dynamic assessment capabilities of IUS, rendering it less suited for nuanced characterization of disease behavior.

Despite these limitations, CT remains irreplaceable in modern Crohn’s disease care. Its unmatched speed and reliability in diagnosing life-threatening complications ensure that it remains the first-line modality in acute presentations. In the acute setting, CT demonstrates sensitivity exceeding 90% for the detection of intra-abdominal abscesses and high-grade obstruction. However, unlike MRE, CT lacks validated activity indices and functional imaging parameters for differentiating inflammatory from fibrotic strictures. Used judiciously and in conjunction with MRE and IUS, CT forms an integral part of a balanced and effective imaging strategy [39,40,41].

## 4. Evolution of ECCO–ESGAR Imaging Guidelines for Crohn’s Disease

Across successive ECCO–ESGAR guideline updates, the role of cross-sectional imaging in Crohn’s disease has evolved toward a more structured, radiation-sparing, and clinically integrated approach [8,9,10].

In the 2013 ECCO–ESGAR imaging consensus [8], MRE was established as the preferred radiation-free alternative to CT, offering comparable diagnostic accuracy for luminal and extramural disease while avoiding cumulative ionizing radiation; pelvic MRI was endorsed as the imaging modality of choice for assessment of perianal fistulizing disease. Intestinal ultrasound (IUS) was recognized as a well-tolerated, non-invasive technique, particularly useful for terminal ileal and colonic disease and for guiding interventional procedures such as abscess drainage, although operator dependency and limitations related to bowel gas and body habitus were acknowledged. CT and CT Enterography (CTE) were highlighted for speed and availability, with CTE favored over CT Enteroclysis for patient acceptance, but radiation exposure was identified as a major drawback, restricting repeated use, despite CT’s importance in acute settings and image-guided interventions.

The 2019 ECCO–ESGAR diagnostic assessment guidelines [9] further embedded imaging into diagnostic algorithms, endorsing MRE and IUS as equivalent first-line options for small-bowel evaluation in newly diagnosed or symptomatic Crohn’s disease, alongside capsule endoscopy. A key refinement was the explicit acknowledgment that the choice between modalities should be guided by local expertise and availability. MRE was emphasized for accurate mapping of disease extent and transmural involvement, supported by comparative evidence such as the METRIC trial, whereas IUS was highlighted for its immediacy, accessibility, and ability to reduce further testing when clearly positive, with enhanced performance using contrast-based techniques (e.g., SICUS or CEUS). In contrast, CTE was increasingly confined to acute or emergency scenarios, with low-radiation protocols considered mainly when other modalities were unavailable, in older patients, or for rapid detection of complications such as perforation, strictures, or abscesses. The 2025 ECCO–ESGAR–ESP–IBUS guideline [10] consolidates these trends and formalizes a “triangle” of endoscopy, IUS, and MRE across the disease course. At initial diagnosis, ileocolonoscopy with biopsies combined with IUS and/or MRE is recommended as first-line, while CTE is positioned as a fallback option in acute settings or when MRI and ultrasound are contraindicated or unavailable. For disease monitoring in a treat-to-target framework, the guideline strongly promotes repeated, non-invasive cross-sectional imaging with IUS or MRE at baseline and early reassessment after therapy initiation or optimization. In the evaluation of complications such as strictures, MRE, IUS, and CTE are acknowledged to have broadly comparable diagnostic performance; however, MRE is preferred when characterization of the fibrotic component is clinically relevant due to superior soft-tissue contrast, while recognizing that no current imaging modality can reliably quantify fibrosis (Table 3 and Table 4). Overall, the 2025 update underscores a patient-centered, radiation-sparing imaging strategy, with CT reserved for specific, time-critical clinical scenarios. This conceptual framework aligns with current ECCO–ESGAR recommendations and treat-to-target strategies, which emphasize transmural assessment and cross-sectional imaging beyond mucosal healing alone.

## 5. Perianal Crohn’s Disease: The Role of MRI and Complementary Techniques

Perianal involvement represents one of the most debilitating manifestations of Crohn’s disease, with a substantial impact on quality of life and long-term prognosis. Fistulas and abscesses in this region are frequently complex, may be recurrent, and often coexist with active luminal disease. Because physical examination is limited in its ability to define the full extent and configuration of fistulous tracts, cross-sectional imaging, particularly MRI, has become indispensable in both baseline assessment and longitudinal follow-up [42,43].

MRI is widely regarded as the gold standard imaging modality for perianal Crohn’s disease, owing to its exquisite soft-tissue contrast and multiplanar capabilities. A typical MRI protocol for perianal fistulas includes high-resolution T2-weighted sequences with and without fat suppression in axial, coronal, and often oblique planes aligned with the anal canal, together with T1-weighted images before and after gadolinium administration. Fat-suppressed post-contrast images are particularly helpful in delineating enhancing fistula tracts and active inflammatory components, while T2-weighted sequences excel at highlighting fluid-rich collections and oedematous tissues. Many centres increasingly add diffusion-weighted imaging (DWI), which may further characterize inflammatory activity and help differentiate between active tracts and more quiescent fibrotic tissue [42,43,44,45,46,47,48,49,50,51].

From a morphological perspective, MRI enables precise classification of fistulas according to established schemes (e.g., Parks’ classification), distinguishing intersphincteric, trans-sphincteric, supra-sphincteric, and extrasphincteric trajectories, as well as identifying secondary extensions and supralevator involvement. This detailed anatomical mapping is fundamental for surgical planning, including the placement of setons, the choice and timing of sphincter-sparing procedures, and the assessment of potential risks to continence. MRI is also highly sensitive for detecting associated abscesses, which may be small, deeply located, and clinically occult. Identifying these collections before initiating or intensifying immunosuppressive therapy is critical to avoid sepsis and treatment failure [42,43,44,45,46,47,48,49,50,51].

The van Assche MRI score, introduced in the early 2000s, was a landmark attempt to quantify perianal disease severity by integrating features such as the number and location of fistula tracts, degree of extension, presence of abscesses, and T2-weighted signal characteristics [47,48]. Although its routine use in everyday practice is variable, the score has been widely adopted in clinical trials and longitudinal studies as a structured way to monitor response to therapies such as anti-TNF agents (Table 5). Subsequent work has proposed modified versions of the van Assche index and alternative MRI-based scoring systems, aiming to improve sensitivity to change and to correlate more closely with clinical and patient-reported outcomes [49]. A significant recent advancement is the MAGNIFI-CD index, a novel MRI-based scoring system specifically designed to refine the evaluation of perianal fistulizing Crohn’s disease by integrating both morphological and inflammatory biomarkers into a unified framework [50]. The MAGNIFI-CD score systematically quantifies active inflammatory features—including mural and perilesional enhancement patterns, diffusion-weighted imaging characteristics, and the presence of inflammatory oedema—alongside established structural descriptors (Table 5). Early validation studies suggest that the MIGNIFI-CD index demonstrates superior sensitivity to change compared with traditional scoring systems and may better predict clinically meaningful outcomes, such as fistula healing or persistence of occult activity despite apparent clinical remission. By incorporating a broader range of MRI parameters and emphasising functional imaging markers, the MIGNIFI-CD index represents a promising tool for standardising radiologic assessment in both routine practice and clinical research, particularly within treat-to-target strategies [51].

MRI plays a crucial role in the evaluation of treatment response in perianal Crohn’s disease. Clinical closure of external openings does not necessarily equate to radiologic healing, as residual active tracts or small collections may persist despite apparently satisfactory external findings. Longitudinal MRI studies have demonstrated that, in patients treated with biologic therapies, perianal fistula tracts often decrease in caliber and contrast enhancement before undergoing complete fibrosis. Persistent T2 hyperintensity or ongoing contrast enhancement has been shown to predict disease relapse, even in the absence of overt clinical drainage [43,44,45,46,47,48,49,50,51]. For this reason, MRI is increasingly being integrated into treat-to-target strategies for perianal Crohn’s disease, with radiological improvement or healing recognized as a key therapeutic endpoint alongside clinical remission.

Other imaging modalities can complement MRI in selected situations. Endoanal ultrasound (EAUS) offers excellent spatial resolution near the anal canal and can be particularly useful in preoperative evaluation of sphincter integrity; however, its field of view is limited and it is less effective for complex or high fistulas. It also requires deep sedation in the presence of inflammatory perianal involvement. CT, by contrast, is generally reserved for acute presentations with suspected pelvic abscesses, sepsis, or other complications but it is clearly inferior to MRI for detailed fistula mapping and is avoided for repeated follow-up because of radiation exposure [51].

In summary, MRI has become the cornerstone of perianal Crohn’s disease imaging. It provides an unparalleled combination of anatomical detail, functional information, and reproducible scoring systems that together guide both medical and surgical decision-making. The integration of MRI findings with clinical assessment, endoscopy, and biomarkers is essential for a truly comprehensive evaluation of this particularly challenging phenotype of Crohn’s disease.

## 6. Discussion

The choice between IUS, MRE, and computed tomography (CT or CT Enterography, CTE) in Crohn’s disease depends strongly on the clinical context, the diagnostic question, and the patient’s need for repeat evaluation over time. Although these modalities can be viewed as complementary, each possesses strengths that make it uniquely suited to particular scenarios in the diagnostic and therapeutic pathway (Table 3, Table 4 and Table 6).

IUS has become a central tool for routine follow-up thanks to its accessibility to clinicians and gastroenterologists specifically, bedside availability, and complete absence of radiation. Its ability to provide real-time information about bowel wall thickness, vascularity, and peristalsis gives clinicians a rapid understanding of inflammatory activity. When performed by experienced operators, either radiologists or gastroenterologists, IUS is valuable for monitoring treatment response, often as part of tight-control strategies. However, its performance is reduced when the operator has limited experience, and even in expert hands it may be inadequate in cases of complex fistulizing disease, when bowel loops are located deep within the abdomen, in obese patients, or when proximal small-bowel involvement is suspected. For these reasons, although IUS is excellent for follow-up and for the early detection of changes in disease activity, it is not sufficient as a standalone modality for comprehensive disease mapping, initial diagnosis, or complete disease staging.

MRE remains the most comprehensive modality for both initial diagnosis and staging or re-staging Crohn’s disease [52,53,54]. Its multiparametric nature allows simultaneous assessment of bowel wall thickness, mural oedema, contrast enhancement patterns, transmural inflammation, mesenteric vascularity, and perienteric inflammatory changes. These features make it especially powerful for characterizing disease activity and for distinguishing inflammatory from fibrotic strictures, an essential task that strongly influences medical versus surgical management. Transmural healing is an emerging and ambitious treatment goal that goes beyond mucosal healing and reflects the concept of deep MRI remission in Crohn’s disease. Multiparametric MRE is one of the most comprehensive non-invasive tools currently available to assess this target [29]. MRE is also the preferred technique for identifying penetrating complications such as sinus tracts, fistulas, and abscesses, particularly when these are located in the mesentery or retroperitoneum, or in the perianal region [53,54]. Because it avoids ionizing radiation, MRE is well suited for patients with long disease duration and for younger individuals who require frequent re-evaluation. It is also the preferred modality in long-term surveillance, especially in patients with longstanding small-bowel disease who are at risk for complications such as small-bowel neoplasia or progressive wall thickening of uncertain significance. The need for radiation-sparing strategies is even more relevant in the pediatric population.

In pediatric Crohn’s disease, imaging strategies should prioritize radiation-free modalities to minimize cumulative lifetime exposure. MRE provides comprehensive evaluation of disease extent and complications but may be limited by longer examination times and the need for patient cooperation, particularly in younger children. Intestinal ultrasound (IUS) is especially advantageous in this setting because it is non-invasive, well tolerated, and easily repeatable, making it suitable for close monitoring and treat-to-target strategies. In addition, ultrasound parameters have shown good diagnostic performance in differentiating inflammatory from fibrotic strictures, supporting therapeutic decision-making. An integrated radiation-sparing approach combining IUS for routine assessment and MRE for comprehensive evaluation appears particularly appropriate in the pediatric population [55,56]. This approach further supports the concept of a personalized imaging strategy based on patient characteristics, clinical scenario, and the need for repeated assessment over time.

The limitations of MRE relate mainly to availability, cost, examination duration, and the need for bowel preparation and patient cooperation.

CT, particularly CT Enterography, retains a critical and irreplaceable role in acute care settings. In the emergency context, where perforation, severe obstruction, abscesses, or other life-threatening complications must be identified quickly, CT is unquestionably the most efficient modality. Its rapid acquisition, extensive availability, and excellent spatial resolution make it the preferred investigation when patients present with severe abdominal pain, fever, systemic toxicity, or suspected sepsis. While CT offers excellent visualization of extramural disease and penetrating complications, its use outside the emergency setting is limited by exposure to ionising radiation, which is a major consideration in the typically young Crohn’s population. As a result, outside of acute situations or cases requiring rapid triage, CT is usually reserved for patients who cannot undergo MRE or when MRE is not available in a clinically acceptable timeframe. In selected cases, particularly in surveillance of unexplained small-bowel wall thickening or when small-bowel polyps are suspected, CT enterography may complement MRE, although this is relatively uncommon (Table 6).

In routine clinical practice, to conclude, the three modalities fall into a clear, though flexible, pattern: IUS is the preferred tool for frequent monitoring and routine follow-up; MRE is the central modality for comprehensive staging, restaging, and evaluation of complications; CT/CTE is indispensable in emergencies or when rapid decision-making is required. In patients with long-standing disease, MRE becomes particularly important during surveillance due to its ability to detect subtle structural changes, while CT may be used selectively when MRE results are inconclusive or when specific luminal abnormalities such as small-bowel polyps require further evaluation. Thus, although these imaging techniques overlap in diagnostic capability, each occupies a distinct role shaped by its strengths and limitations. Their integrated use forms the foundation of modern, individualized imaging strategies in Crohn’s disease.

## Figures and Tables

**Table 1:** MRE routine protocols using different Magnetic Field Strength.

1.5T Magnet (Avanto, Siemens)	3T Magnet (Discovery, GE)
HASTE on axial/coronal planes (Half Fourier Acquisition Single Shot Turbo Spin Echo)	SSFSE (Single Shot Fast Spin Echo)
TrueFISP (True Fasting Imaging Steady-State free Precession sequence)	FIESTA (Fast Imaging Employing STeady-state Acquisition)
Optional: motility imaging	Optional: motility imaging
DWI (b values: 50, 500, 1000) Diffusion Weighted Imaging	DWI (b values: 50, 500, 1000) Diffusion Weighted Imaging
BLADE multi-shot Turbo Spin Echo (TSE) sequence	PROPELLER multi-shot Turbo Spin Echo (TSE) sequence
SINGLE SHOT T2W FS Thick-Slab	SINGLE SHOT T2W FS Thick-Slab
VIBE pre post Gadolinium injection: T1-weighted Volumetric Interpolated Breath-hold Examination	LAVA (Liver Acquisition with Volume Acquisition) pre post Gadolinium injection: T1-weighted 3D Fast Spoiled Gradient Echo sequence

**Table 2:** Comparison of different MRI Crohn’s Disease Activity Scores.

	MARIA Score *	Clermont Score	CDMI (Crohn’s Disease MRI Index)
Full Name	Magnetic Resonance Index of Activity	Clermont Score	Crohn’s Disease MRI Index
Primary Purpose	Quantitative assessment of inflammatory activity on MRI	Quantitative assessment of inflammatory activity using DWI-MRI	MRI-based activity index for Crohn’s disease
Imaging Basis	Contrast-enhanced MRI	Diffusion-weighted imaging (DWI) MRI (no contrast required)	Contrast-enhanced MRI
Use of Gadolinium Contrast	Required	Not required	Required
Key Parameters Included	- Bowel wall thickness (mm) - Relative contrast enhancement (RCE) - Presence of edema - Presence of ulcerations	- Bowel wall thickness (mm) - Apparent diffusion coefficient (ADC) - Presence of ulcers	- Bowel wall thickness - T2 signal intensity (edema) - Contrast enhancement - Presence of complications
Validation	Correlated with endoscopic severity (e.g., CDEIS)	Validated against MARIA and endoscopic indices	Correlated with endoscopic findings
Segmental vs. Global Assessment	Segmental (per bowel segment)	Segmental	Segmental
Strengths	- Widely validated - Strong correlation with endoscopy - Good reproducibility	- Avoids gadolinium - Useful in patients with renal impairment - Faster protocol	- Simpler structure - Incorporates transmural and extramural features
Limitations	- Requires contrast - Longer acquisition time	- Requires high-quality DWI - ADC variability between scanners	- Less widely validated - Greater interobserver variability

* Note: a Simplified MaRIA (sMarRIA) has also been proposed, which is easier to use in clinical practice, as it avoids Gadolinium and uses simpler formulas [25].

**Table 3:** Strengths and Limitations of Key Imaging Modalities in Crohn’s Disease.

Feature	IUS	MRE	CT
Radiation	None	None	Yes
Visualization of entire small bowel	Moderate	Excellent	Good
Extramural disease	Moderate	Excellent	Excellent
Assessment of activity	Good	Excellent	Good
Fibrosis assessment	Limited	Best available non-invasive tool	Limited
Fistulas/Abscesses	Moderate	Excellent	Excellent
Use in emergency	Limited	Limited	Best
Repeatability	Excellent	Excellent	Poor

**Table 4:** Recommended Imaging Modality by Clinical Scenario.

Clinical Scenario	Preferred Modality	Justification
Initial diagnosis	MRE + IUS	Radiation-free; high diagnostic yield; complementary luminal + extraluminal information
Monitoring treatment response	IUS ± MRE	IUS for frequent bedside assessment; MRE for full reassessment when needed
Suspected obstruction	CT or MRE	CT best in emergencies; MRE preferred for elective evaluation or characterization of stricture phenotype
Suspected abscess	CT or MRE	CT for acute settings; MRE for detailed elective evaluation
Perianal disease	High-resolution MRI of the pelvis (HRMRI)	Gold standard for fistulas, abscesses, and sphincter complex
Suspected penetrating disease	MRE	Superior assessment of sinus tracts, fistulas, and extramural spread
Routine follow-up	IUS	Accessible, radiation-free, low cost, repeatable
Staging/Restaging	MRE	Best technique for full mapping of small bowel and complications
Surveillance in long-term disease	MRE/CTE	MRE preferred; CTE only when MRE unavailable or for detailed evaluation of small-bowel polyps or abnormal wall thickening

**Table 5:** Comparison of MRI Scores for assessment of perianal fistula activity/severity.

	MAGNIFI-CD Score	Van Assche Score [47,49]
Full Name	Magnetic Resonance Index of Fistula Inflammation in Crohn’s Disease	Van Assche MRI Score for Perianal Fistulas
Primary Purpose	Quantitative assessment of inflammatory activity in perianal fistulas	Assessment of anatomical complexity and inflammatory severity of perianal fistulas
Imaging Basis	Pelvic MRI (T2-weighted and post-contrast T1-weighted sequences)	Pelvic MRI (T2-weighted)
Use of Gadolinium Contrast	Required (for enhancement assessment)	Not required
Main Parameters Included	- Dominant fistula tract length - Hyperintensity on T2-weighted images - Contrast enhancement - Presence and size of abscesses - Presence of secondary tracts	- Number of fistula tracts - Location (intersphincteric, transsphincteric, etc.) - Extension (supralevator, extrasphincteric) - T2 hyperintensity - Abscess presence - Rectal wall involvement
Validation	Correlated with clinical response and treatment outcomes	Correlated with clinical severity and used in therapeutic trials
Strengths	- Specifically designed for inflammatory activity monitoring - Differentiation between active inflammation vs. fibrosis - Higher prognostic value for fistula healing	- Widely used in clinical trials - Established reproducibility
Limitations	- Requires contrast - Relatively recent, less long-term validation	- Limited sensitivity to subtle inflammatory changes - May not fully reflect treatment-induced activity changes

**Table 6:** Imaging Algorithm for Crohn’s Disease.

Clinical Scenario	First-Line Imaging	Why	When to Escalate
Suspected Crohn’s disease	MRE (preferred) or IUS (expert center only)	Detects small-bowel disease, transmural inflammation, strictures, and complications	Use CTE if MRI unavailable, contraindicated, or urgent
Younger/pediatric patients			
Known CD—mild/moderate symptoms or routine monitoring	IUS	Point-of-care assessment of bowel wall thickness, Doppler activity, mesenteric inflammation; radiation-free	Escalate to MRE if proximal small bowel not visualized, strictures suspected, or full disease mapping needed
Suspected complication (severe pain, fever, obstruction, sepsis, mass-polyps)	CT abdomen/pelvis ± CT Enterography	Faster, widely available, best for abscess, perforation, obstruction, phlegmon	Use MRE for stable patients, detailed mapping, or follow-up without radiation
Suspected stricturing disease	MRE	Defines length, severity, pre-stenotic dilation, and penetrating features	Add IUS for follow-up or CT if acute obstruction
Perianal symptoms (pain, drainage, fistula)	Pelvic HR MRI	Gold standard for fistula anatomy, abscesses, and treatment planning	—

## Data Availability

No new data were created or analyzed in this study. Data sharing is not applicable to this article.
